# Risk factors for level V metastasis in patients with N1b papillary thyroid cancer

**DOI:** 10.1186/s12957-022-02782-0

**Published:** 2022-09-30

**Authors:** Jin Gu Kang, Jung Eun Choi, Su Hwan Kang

**Affiliations:** Department of Surgery, Yeungnam University College of Medicine, 170 Hyunchoong-ro, Namgu, Daegu, 42415 South Korea

**Keywords:** Lymph node metastasis, Neck dissection, Thyroid cancer

## Abstract

**Background:**

Papillary thyroid carcinoma (PTC) is the most common type of thyroid cancer, and its incidence has increased. Lateral lymph node metastasis (LLNM) implies a worse prognosis than central lymph node metastasis, with a higher recurrence rate and decreased disease-free survival. The 2015 American Thyroid Association guidelines recommend compartmental node dissection in patients with LLNM to reduce the risk of recurrence and mortality. The purpose of this study was to identify the risk factors for level V lymph node (LN) metastasis in patients with N1b papillary thyroid cancer (PTC).

**Methods:**

A total of 110 consecutive patients who underwent total thyroidectomy with lateral neck dissection for PTC between April 2016 and April 2022 were retrospectively enrolled. Based on level V metastasis, 94 patients were divided into two groups, and their clinicopathological characteristics were compared. Univariable analysis were used to assess the factors associated with level V metastasis. Spearman correlation analysis were used to assess the correlation between tumors and LN. The receiver operating characteristic (ROC) curve was used to determine the optimal cutoff value for the number of metastatic LNs at each level for level V metastasis.

**Results:**

The number of metastatic LNs and lymph node ratio (LNR) in level II were significantly associated with level V metastasis (*P* = 0.011 and 0.001, respectively). The number of metastatic LNs in level II and those in the total number of levels correlated with the number of metastatic LNs in level V (rho = 0.331, 0.325, and *P* = 0.001, 0.001, respectively). The cutoff value for the number of metastatic LNs in level II was defined as 2.5 (area under the curve = 0.757, sensitivity = 50%, specificity = 82.5%, 95% confidence interval [CI] 0.626–0.889, *P* = 0.002). Simultaneous 3-level metastasis (level II, III, and IV) and 3-level with ≥ 2.5 metastatic LNs in level II were significantly associated with level V metastasis (*P* = 0.003 and 0.002).

**Conclusions:**

The number of metastatic LNs and LNR in level II, simultaneous 3-level metastasis (level II, III, and IV), and 3-level with ≥ 2.5 metastatic LNs in level II were significantly associated with level V metastasis. (*P* = 0.011, 0.001, 0.003, and 0.002, respectively). In the future, larger-scale multi-institutional studies were needed to find out predictors for level V metastasis.

**Supplementary Information:**

The online version contains supplementary material available at 10.1186/s12957-022-02782-0.

## Background

Papillary thyroid carcinoma (PTC) is the most common type of thyroid cancer, and its incidence has increased. PTC has an excellent prognosis, but it commonly spreads to the central neck compartment (VI and VII) [1]. N1b was defined as metastasis to lateral cervical or retropharyngeal or superior mediastinal lymph nodes [2]. LLNM implies a worse prognosis than central LN metastasis, with a higher recurrence rate and decreased disease-free survival [3]. Regional metastases occur first in the central neck compartment and subsequently in the lateral compartment. However, sometimes there is an unexpected LNM pattern. Skip metastasis is defined as a discontinuous lymphatic spread [4]. Skip metastasis to the lateral neck LNs without CLNM was found in 8.7 to 18.9% of PTC with LLNM [5]. It is well established that patients with LNM have a higher risk of recurrence; however, the effect of LNM on survival remains debatable.

The 2015 American Thyroid Association guidelines recommend compartmental node dissection in LLNM because of the risk of recurrence and mortality [2]. “Berry picking” is defined as a limited lymphadenectomy that involves the removal of enlarged or suspicious LNs only. Berry picking is no longer used as a treatment because of the high incidence of recurrence. The optimal extent of surgery for LLNM is relatively well-established. Modified radical neck dissection (MRND) is considered the standard treatment and is defined as the removal of all the lateral LNs, including levels II–V, with preservation of the internal jugular vein, sternocleidomastoid muscle, and spinal accessory nerve [6]. Level V dissection can cause serious complications such as shoulder dysfunction due to injury of the spinal accessory nerve. It can also cause other clinically important morbidities, such as neck numbness due to injury to the supraclavicular nerves [7]. Selective lymph node dissection (SLND) is defined as a functional neck dissection with the removal of less than all nodal levels and preservation of non-lymphatic structures [8]. The most common levels involved in SLND are levels III and IV [9, 10]. One study demonstrated that MRND is too aggressive, and the incidence of metastatic LNs is lower in levels II and V than in levels III and IV [11].

It is difficult to predict level V metastasis using only previously reported predictors in real-world situations. There is little preoperative evidence, such as tumor size, extrathyroidal extension, or multi-level involvement by ultrasonography (USG). The purpose of this study was to identify the risk factors for level V lymph node (LN) metastasis in patients with N1b papillary thyroid cancer (PTC).

## Methods

### Patient selection

A total of 110 consecutive patients who underwent total thyroidectomy with lateral neck dissection for PTC between April 2016 and April 2022 were retrospectively enrolled. This study was approved by the Yeungnam University Hospital institutional review board. The inclusion criteria were as follows: (1) PTC with LLNM proven by preoperative fine needle aspiration cytology. (2) Dissection of levels II through V with preservation of the spinal accessory nerve, internal jugular vein, and sternocleidomastoid muscle. (3) Description of the largest metastatic focus in LNs by pathology. The exclusion criteria were as follows: (1) less than a modified radical neck dissection (levels II, III, IV, and V). (2) Omission of level V dissection. (3) Insufficient or uncertain number of harvested LNs in any level. All patients were confirmed to have PTC with LLNM based on postoperative pathology results. Finally, 94 patients were enrolled in the study.

### Clinicopathological variables

All patients were evaluated for age, sex, tumor, and LN sizes preoperatively, LN to tumor size ratio, tumor location, bilaterality, multi-focality, extrathyroidal extension (ETE), extranodal extension, stage, size of the largest metastatic focus in the LNs, number of metastatic and harvested LNs, and lymph node ratio (LNR). The tumor size determined using USG was defined as the maximal diameter of the tumor. Tumor location was divided into upper, middle, and lower based on preoperative USG findings. Tumor location and size were evaluated by the largest tumor in the cases of multi-focality. ETE was defined as a gross extrathyroidal extension. Bilaterality, multi-focality, and ETE were confirmed using postoperative pathology results. LN size determined using USG was defined as the maximal diameter of the suspicious LN in any level. The LNR for each level was calculated by dividing the number of metastatic LNs by the number of harvested LNs. The LN size to tumor size ratio was calculated to assess the correlation between tumor size and LN size. Based on level V metastasis, the 94 patients were divided into two groups.

### Assessment of LN

LN levels were defined according to the agreed nomenclature of the 2015 American Thyroid Association guidelines. Level III is arrayed along the jugular vein and bounded by the level of the hyoid bone superiorly and the cricoid cartilage inferiorly. Level II is above level III and level IV below level III. Level V is defined as the posterior triangle lateral to the lateral edge of the sternocleidomastoid muscle [2]. Ipsilateral or bilateral level VI dissection was performed when central node involvement was suspected. All lateral LNs were classified into appropriate levels in the operative field and then permanently sent to the pathologist. Postoperative LN size was defined as the size of the largest metastatic focus in the LNs. The LNR for each level was calculated by dividing the number of metastatic LNs by the number of harvested LNs. Data regarding the involved levels and number of metastatic LNs in each level were collected. The number of involved levels, except level V, was divided into single- and multi-level metastases. Skip metastasis was defined as LLNM without level VI metastasis.

### Follow-up and recurrence

All patients received radioactive iodine treatment and were followed up with physical examination, laboratory tests, and USG at 6 to 12 months intervals. Recurrence of LNs was defined as metastatic LNs that newly appeared after the initial surgery. Fine needle aspiration cytology, with or without fine needle aspiration thyroglobulin, was performed when LN recurrence was suspected.

### Statistical analysis

Continuous variables are presented as mean ± standard deviation (SD), and categorical variables are presented as numbers and percentages. In univariable analysis, the independent *t*-test, chi-square test, and Fisher’s exact test were used to assess the factors associated with level V metastasis. Spearman correlation analysis was used to assess the correlation between tumors and LN, and they are presented as coefficients of correlation and *P* values. The receiver operating characteristic (ROC) curve was used to determine the optimal cutoff value for the number of metastatic LNs at each level for level V metastasis. All statistical analyses were performed using SPSS version 21.0 (SPSS, Inc., Chicago, IL, USA). Statistical significance was set at *P* values < 0.05.

## Results

### Baseline characteristics

The clinicopathological characteristics of the 94 patients are presented in Table 1. A total of 94 patients were analyzed, of whom 26 (27.7%) were men and 68 (72.3%) were women, with a mean age of 44.80 ± 13.18 years. All patients underwent total thyroidectomy with MRND and postoperative radioiodine therapy. Ten patients (10.6%) underwent bilateral MRND. Postoperative pathological examination showed PTC with LLNM in all the patients. Tumor size and the largest size of LN measured using USG were 16.02 ± 10.16 mm and 14.66 ± 7.35 mm, respectively. LN size to tumor size ratio calculated using the data from USG was 1.23 ± 0.92 mm. Multi-level suspicious LNs evaluated using USG were found in 43 (45.7%) patients. Tumors were observed in 35 (37.2%), 57 (60.6%), and 2 (2.1%) patients in the upper, middle, and lower poles, respectively. Gross ETE, bilaterality, and multi-focality were observed in 10 (10.6%), 37 (39.4%), and 50 (53.2%) patients, respectively. Tumor size was 15.06 ± 8.12 mm, the largest metastatic focus in LN was 12.98 ± 8.76 mm, and metastatic focus size to tumor size ratio was 1.07 ± 0.89. Among the 94 patients, 71 (75.5%) patients were in stage I, and 23 (24.5%) patients were in stage II. There were 70 (74.5%) patients in T1, 13 (13.8%) in T2, and 11 (11.7%) in T3. There were four recurrences, which included two in level III at 12 months after surgery, one in the retropharyngeal region after 14 months, and one in level II after 24 months. The mean follow-up period was 33.44 ± 21.1 months.

**Table 1:** Clinicopathologic characteristics

Characteristic	Value (*n* = 94)
Gender	
Male	26 (27.7)
Female	68 (72.3)
Age (year)	44.80 ± 13.18
Tumor size by USG (mm)	16.02 ± 10.16
The largest size of LN by USG	14.66 ± 7.35
LN/tumor size ratio by USG	1.23 ± 0.92
Multi-level suspicious LNs by USG	43 (45.7)
Tumor location	
Upper	35 (37.2)
Middle	57 (60.6)
Lower	2 (2.1)
Bilaterality	37 (39.4)
Extrathyroidal extension	10 (10.6)
Multifocality	50 (53.2)
Tumor size by pathology (mm)	15.06 ± 8.12
The largest metastatic focus of LN by pathology (mm)	12.98 ± 8.76
Metastatic focus/tumor size ratio by pathology	1.07 ± 0.89
MRND	
Right	48 (51.1)
Left	36 (38.3)
Both	10 (10.6)
T stage	
TI	70 (74.5)
T2	13 (13.8)
T3	11 (11.7)
Stage	
I	71 (75.5)
II	23 (24.5)
Recurrence	4 (4.3)
Follow-up period (months)	33.44 ± 21.1

Values are presented as number (%) or mean ± standard deviation

*USG* ultrasonography, *LN* lymph node, *MRND* modified radical neck dissection

### Lymph node metastasis in each level

The metastatic LNs and LNR in each level are shown in Table S1. We calculated the number of metastatic and harvested LNs by using average number. The number of total metastatic and harvested LNs was 10.62 ± 7.34 and 40.98 ± 15.86, respectively. The number of metastatic LNs in each level was 1.47 ± 1.85 in level II, 2.21 ± 2.35 in level III, 1.50 ± 1.64 in level IV, 0.19 ± 0.53 in level V, and 5.27 ± 4.89 in level VI. LNR at each level was as follows: 0.27 ± 0.17 in total, 0.27 ± 0.32 in level II, 0.26 ± 0.24 in level III, 0.21 ± 0.23 in level IV, 0.04 ± 0.15 in level V, and 0.52 ± 0.37 in level VI. Single-level LN metastasis, except in level V, was found in 26 (27.7%) patients (7 (7.4%) in level II, 15 (16%) in level III, and 4 (4.3%) in level IV). Multi-level LN metastasis, except level V, was found in 68 (72.3%) patients (12 (12.8%) in levels II and III, 7 (7.4%) in levels II and IV, 22 (23.4%) in levels III and IV, and 27 (28.7%) in levels II, III, and IV). Skip metastasis (LLNM without level VI metastasis) was found in 15 patients (16.0%) as follows: 2 (13.3%) in level II, 5 (33.4%) in level III, 1 (6.7%) in level IV, 2 (13.3%) in levels II and III, 2 (13.3%) in levels II and IV, 2 (13.3%) in levels III and IV, and 1 (6.7%) in levels II, III, and IV.

### Clinicopathologic factors associated with level V metastasis

Table 2 shows the factors associated with level V metastasis in the two groups. Univariable analysis showed that there was no difference in age, sex, tumor size, the largest LN size, LN to tumor size ratio, tumor location, multi-focality, bilaterality, ETE, tumor size (pathology), metastatic focus to tumor size ratio (pathology), extranodal extension, stage, and single-level metastasis except level V. However, multi-level suspicious LNs (USG), largest metastatic focus of LN (pathology), multi-level (level II, III, and IV) metastasis, number of metastatic LNs in level II, LNR in level II, 3-level metastasis with ≥ 2.5 metastatic LNs in level II, and less than 3-level metastasis with ≤ 2 metastatic LNs in level II were significantly associated with level V metastasis (*P* = 0.037, 0.039, 0.003, 0.011, 0.001, 0.002, and 0.004, respectively).

**Table 2:** Univariate analysis associated to the level V metastasis

Variable	Univariate		
	Lv5 (+) (*n* = 14)	Lv5 (−) (*n* = 80)	*P* value
Age (year)	40.21 ± 9.34	45.6 ± 13.63	0.159
Gender			1.000
Male	4 (28.6)	22 (38)	
Female	10 (71.4)	58 (62)	
Tumor size (USG)	20.29 ± 11.97	15.28 ± 9.7	0.089
The largest LN size (USG)	15.21 ± 3.91	14.56 ± 7.81	0.635
LN/tumor size ratio (USG)	1.07 ± 0.77	1.26 ± 0.94	0.485
Multi-level suspicious LNs (USG)	10 (71.4)	33 (41.3)	0.037
Tumor location			0.549
Upper	7 (50)	28 (35)	
Middle	7 (50)	50 (62.5)	
Lower	0	2 (2.5)	
Multifocality	9 (64.3)	41 (51.3)	0.367
Bilaterality	6 (42.9)	31 (38.8)	0.772
Extrathyroidal extension	3 (21.4)	7 (8.8)	0.167
Tumor size (pathology)	19.57 ± 11.93	14.28 ± 7.06	0.128
Size of the largest metastatic focus (pathology)	17.43 ± 8.28	12.2 ± 8.66	0.039
Metastatic focus/tumor size ratio (pathology)	1.19 ± 0.96	1.05 ± 0.88	0.584
Extranodal extension	10 (71.4)	42 (52.5)	0.189
Stage			0.505
I	12 (85.7)	59 (73.8)	
II	2 (14.3)	21 (26.2)	
CLNM	14 (100)	65 (81.3)	0.116
Single level metastasis (except level V)			
Level II	2 (14.3)	5 (6.3)	0.279
Level III	0	15 (18.8)	0.116
Level IV	1 (7.1)	3 (3.8)	0.481
Multi-level metastasis (except level V)			
Level II and III	2 (14.3)	10 (12.5)	1.0
Level II and IV	0	7 (8.8)	0.589
Level III and IV	0	22 (27.5)	0.035
Level II, III, and IV	9 (64.3)	18 (22.5)	0.003
Number of metastatic LNs			
Total	16.93 ± 8.06	9.51 ± 6.66	< 0.001
Level II	3.07 ± 2.34	1.19 ± 1.61	0.011
Level III	3.93 ± 4.29	1.91 ± 1.69	0.105
Level IV	1.93 ± 1.64	1.43 ± 1.64	0.293
Level VI	7.00 ± 4.19	4.96 ± 4.96	0.151
LNR			
Total	0.35 ± 0.16	0.25 ± 0.17	0.036
Level II	0.52 ± 0.35	0.22 ± 0.29	0.001
Level III	0.32 ± 0.23	0.25 ± 0.24	0.330
Level IV	0.32 ± 0.32	0.19 ± 0.20	0.158
Level VI	0.58 ± 0.32	0.51 ± 0.38	0.539
3-level with ≥ 2.5 metastatic LNs in level II	6 (42.9)	6 (7.5)	0.002
Less than 3-level with ≤ 2 metastatic LNs in level II	4 (28.6)	55 (68.8)	0.004

Values are presented as mean ± standard deviation, number only, or number (%)

*OR* odds ratio, *CI* confidence interval, *USG* ultrasonography, *LN* lymph node, *CLNM* central lymph node metastasis, *LNR* lymph node ratio

### Correlation between tumor size and LN

The correlation between tumor size and LN is shown in Table 3. Tumor size correlated with the total number of metastatic LNs and the number of metastatic LNs in levels II and VI (rho = 0.297, 0.232, and 0.238, and *P* = 0.004, 0.025, and 0.021, respectively). The number of metastatic LNs in level II and total levels correlated with the number of metastatic LNs in level V (rho = 0.331, 0.325, and *P* = 0.001, 0.001, respectively).

**Table 3:** Correlation analysis between tumor and metastatic LNs

Independent variable	Dependent variable	Coefficient of correlation (rho)	*P* value
Tumor size (pathology)	LN size (pathology)	0.136	0.191
	Total number of metastatic LNs	0.297	0.004
	Number of metastatic LNs, level II	0.232	0.025
	Number of metastatic LNs, level III	0.067	0.524
	Number of metastatic LNs, level IV	0.164	0.114
	Number of metastatic LNs, level V	0.175	0.092
	Number of metastatic LNs, level VI	0.238	0.021
Number of metastatic LNs, level II	Number of metastatic LNs, level V	0.331	0.001
Number of metastatic LNs, level VI	Number of metastatic LNs, level V	0.198	0.055
Total number of metastatic LNs	Number of metastatic LNs, level V	0.325	0.001

*LN* lymph node

### Optimal cutoff of the number of metastatic LNs in level II related to level V metastasis

**Fig. 1:**
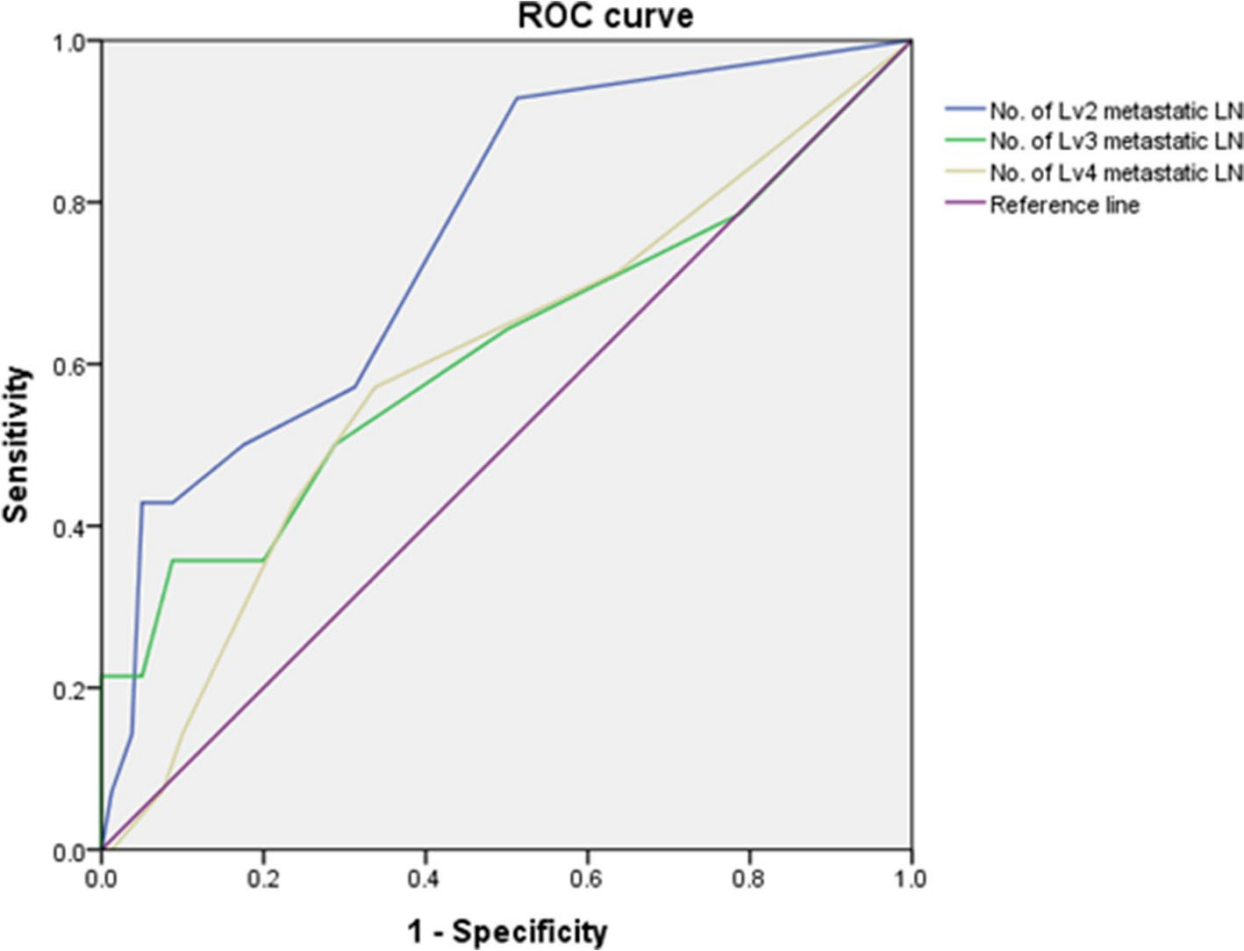
Receiver operating characteristic (ROC) curve according to the number of metastatic lymph nodes in each level related to the level V metastasis

In univariable analysis, the number of metastatic LNs and LNR in level II was significantly associated with level V metastasis (*P* = 0.011 and 0.001, respectively). The number of metastatic LNs in level II was not an independent factor for level V metastasis, but it has the potential to be a co-factor based on 3-level with ≥ 2.5 metastatic LNs in level II and less than 3-level with ≤ 2 metastatic LNs in level II (*P* = 0.002 and *P* = 0.004, respectively). Furthermore, the number of metastatic LNs in level II correlated with the number of metastatic LNs in level V (rho = 0.331, *P* = 0.001) (Table 3). As shown in Fig. 1, ROC curve analysis was used to assess the value of the number of metastatic LNs in each level for level V metastasis. The cutoff value for the number of metastatic LNs in level II was defined as 2.5 (AUC = 0.757, sensitivity = 50%, specificity = 82.5%, 95% CI 0.626–0.889, *P* = 0.002).

## Discussion

The current standard treatment for N1b PTC is MRND includes the removal of LNs from level II to V [12, 13]. One meta-analysis study included 23 papers and demonstrated that histological level V metastasis was found in 287 of 1272 (22.5%) patients who underwent level V dissection [14]. Other studies have shown that the incidence of level V metastasis ranges between 20% and 53% [15, 16]. In contrast, one study reported that the occult metastatic rate in level V was 6.4%, and the lateral neck dissection group did not show a higher disease-free survival rate than that seen in the control group [6]. Because of the lower complication rate, lower incidence rate, and no higher disease-free survival rate associated with lateral neck dissection, level V dissection remains controversial. MRND has a higher risk of injury to the spinal accessory, phrenic, vagus, greater auricular, cervical cutaneous, and supraclavicular nerves. Among these, level V dissection is closely related to spinal accessory nerve and supraclavicular nerve injuries, which can lead to impaired shoulder abduction and numbness of the lateral neck. As the incidence of level V metastasis cannot be ignored, level V dissection is considered essential despite the risk of morbidity. According to the American Thyroid Association guidelines, comprehensive lateral neck dissection includes levels IIA, III, IV, and VB in PTC with LLNM. Compartmental dissection is recommended over berry picking because of the risk of recurrence. Some studies have reported internal jugular node dissection in low-risk PTC with LNM. (levels II, III, and IV) to reduce postoperative complications [11, 17]. Super-selective neck dissection can be used to remove clinically suspicious lateral LNs in levels III and IV without extending the incision. Some studies have demonstrated that MRND has a higher risk of morbidity than selective neck dissection. Moreover, there was no significant benefit in the survival rate compared with selective neck dissection [9, 18]. One study reported that occult metastasis was found in 25% of the level II LNs, and the negative predictive value of fine needle aspiration of level V LNs was approximately 98%. They suggested that super-selective neck dissection may be performed for a single LNM in level III or IV and not for upper location tumors [19].

Suspicious cervical LNs were identified using preoperative USG in 20–31%. The USG features of metastatic LNs include loss of the fatty hilum, round shape, hyperechogenicity, cystic change, calcification, and peripheral vascularity. The USG feature of an absence of a hilum showed high sensitivity (100%) and low specificity (29%). The presence of microcalcifications had the highest specificity [2]. Identification of directly suspicious level V LNs using USG would be the most reliable method. However, the identification rate of level V metastasis using USG was relatively low. Some studies have reported that preoperative USG had low sensitivity (51–62%) and high specificity (79–98%) in the detection of LNM [20, 21]. Neck lymphatic metastasis of PTC occurred mainly in the deep neck lymphatic chain. The lateral compartments are divided into five compartments (levels I through V), and some of them are subsequently divided into subgroups, such as levels IA and IB, IIA and IIB, and VA and VB. LNM occurred in the ipsilateral paratracheal LN and then in the contralateral ones. The lymphatic spread subsequently reaches levels IIA, III, IV, and VB. One meta-analysis showed that 20.9% of the patients had LLNM [22]. Skip metastasis to the lateral LN was reported in approximately 8.7–21.8% [23]. A meta-analysis reported that most LLNM occurred in level II through IV, in the range of 27–65% in level II, 57–82% in level III, and 41–82% in level IV [24]. In the current study, there were 15% LLNM and 16% skip metastasis (LLNM without CLNM). There is little data on the incidence of occult LLNM. In one study, occult LLNM was found in 18.6% of patients. The most common levels of occult LLNM were levels III, IIA, and IV. Occult LLNM was found in level VB (5.7%) but was absent in level VA [16].

Studies on predictive factors for level V metastasis have been conducted. One study reported that tumor size ≥2.5 cm, number of CLNM ≥3, level III metastases, and B-type Raf kinase (BRAF) mutation^V600E^ were independent predictors of level V metastasis [10]. Another study reported that CLNM was an independent factor for LLNM (OR 7.64, 95% CI 5.59–10.44) [25]. In a meta-analysis, male sex, tumor location (upper), tumor size, multi-focality, bilaterality, LVI, ETE, and CLNM were found to be significant risk factors for LLNM [22]. Another study showed that simultaneous 3-level metastasis in LNM was an independent factor for level V metastasis (OR = 8.6, 95% CI 1.42–51.72, *P* = 0.02). Prophylactic level V dissection may only be recommended for N1b PTMC with simultaneous 3-level metastasis [6]. However, whether level V dissection in N1b PTC patients may be omitted with known predictors alone in real-world situations is unclear. For example, if there are more than 3 cm tumors in the upper pole or three suspicious LNs in level VI on USG, it is difficult to decide whether level V dissection should be performed. Furthermore, we obtain limited information regarding ETE and extranodal extension preoperatively. Therefore, we focused on the risk factors and observed two meaningful results. First, simultaneous level II, III, and IV metastasis was significant risk factor for level V metastasis (*P* = 0.003). Second, the number of metastatic LNs in level II correlated with the number of metastatic LNs in level V (rho = 0.331, *P* = 0.001).

LNR is generally calculated by dividing the number of metastatic LNs by the total number of harvested LNs. LNR indicates the quality of surgery or completeness of LN dissection. In one analysis, LNR was a strong determinant of disease-specific mortality, with a threshold LNR of 0.42 [26]. In our study, LNR was calculated as 0.27 ± 0.17 in total, 0.27 ± 0.32 in level II, 0.26 ± 0.24 in level III, 0.21 ± 0.23 in level IV, 0.04 ± 0.15 in level V, and 0.52 ± 0.37 in level VI. LNR in level II and total levels were significantly associated with level V metastasis (*P* = 0.001 and 0.036, respectively). In the current study, we hypothesized that a large tumor size will affect metastatic LN size and level V metastasis. However, tumor size and LN to tumor size ratio were not associated with level V metastasis (*P* = 0.128 and 0.584, respectively). Tumor size did not correlate with LN size (rho = 0.136, *P* = 0.191). There was no significant association between LN to tumor size ratio and level V metastasis (*P* = 0.584). However, we found that tumor size correlated with the number of metastatic LNs in levels II and VI (rho = 0.232, 0.238, and *P* = 0.025, 0.021, respectively).

Our study has some limitations. First, multi-variable analysis was excluded because of inappropriate statistics with small number of cases. Second, the information regarding preoperative USG findings at each level was not sufficient because preoperative USG was performed in different hospitals. Third, this study enrolled a small population of 94 patients, with a short follow-up period. In the future, a multi-center prospective study should be conducted to evaluate the quality of life and survival.

## Conclusions

Our study showed that tumor size correlated with the total number of metastatic LNs and the number of metastatic LNs in levels II and VI (rho = 0.297, 0.232, and 0.238, and *P* = 0.004, 0.025, and 0.021, respectively). The number of metastatic LNs in level II and total levels correlated with the number of metastatic LNs in level V (rho = 0.331, 0.325, and *P* = 0.001, 0.001, respectively). The number of metastatic LNs and LNR in level II, simultaneous 3-level metastasis (level II, III, and IV), and 3-level with ≥ 2.5 metastatic LNs in level II were significantly associated with level V metastasis. (*P* = 0.011, 0.001, 0.003, and 0.002, respectively). In the future, larger-scale multi-institutional studies were needed to find out predictors for level V metastasis.

## Supplementary Information

**Additional file 1: Table S1.** Lymph node metastasis according to each level.

## Data Availability

The datasets used and/or analyzed during the current study are available from the corresponding author on reasonable request.

## Abbreviations

CLNM: Central lymph node metastasis
LLNM: Lateral lymph node metastasis
LNR: Lymph node ratio
MRND: Modified radical neck dissection
PTC: Papillary thyroid cancer
