# Supplementary material for: Risk factors for level V metastasis in patients with N1b papillary thyroid cancer
Source: World J Surg Oncol. 2022 Sep 30;20:327. doi: 10.1186/s12957-022-02782-0 (PMC9524026; doi:10.1186/s12957-022-02782-0)
Supplement: Supplementary file 1 — Additional file 1: Table S1. Lymph node metastasis according to each level. [file 12957_2022_2782_MOESM1_ESM.docx]

**Table S1** Lymph node metastasis according to each level

| Variable | Value |
| --- | --- |
| Total number of metastatic LNs | 10.62 ± 7.34 |
| Total number of harvested LNs | 40.98 ± 15.86 |
| LNR (total) | 0.27 ± 0.17 |
| Number of metastatic LNs |  |
| Level II | 1.47 ± 1.85 |
| Level II (LNR) | 0.27 ± 0.32 |
| Level III | 2.21 ± 2.35 |
| Level III (LNR) | 0.26 ± 0.24 |
| Level IV | 1.5 ± 1.64 |
| Level IV (LNR) | 0.21 ± 0.23 |
| Level V | 0.19 ± 0.53 |
| Level V (LNR) | 0.04 ± 0.15 |
| Level VI | 5.27 ± 4.89 |
| Level VI (LNR) | 0.52 ± 0.37 |
| Skip metastasis (LLNM without CLNM) | 15 (16.0) |
| Single level metastasis (except Lv5) | 26 (27.7) |
| Level II | 7 (7.4) |
| Level III | 15 (16) |
| Level IV | 4 (4.3) |
| Multi-level metastasis (except Lv5) | 68 (72.3) |
| Level II and III | 12 (12.8) |
| Level II and IV | 7 (7.4) |
| Level III and IV | 22 (23.4) |
| Level II, III, and IV | 27 (28.7) |

Values are presented as mean±standard deviation or number (%)

*LN* Lymph node, *LNR* Lymph node ratio, *LLNM* Lateral lymph node metastasis, *CLNM* Central lymph node metastasis
